# Supplementary material for: Dolomite luminescence thermochronometry reconstructs the low-temperature exhumation history of carbonate rocks in the central Apennines, Italy
Source: Commun Earth Environ. 2025 Apr 2;6(1):252. doi: 10.1038/s43247-025-02216-1 (PMC11964923; doi:10.1038/s43247-025-02216-1)
Supplement: Supplementary file 2 — supplementary materials [file 43247_2025_2216_MOESM2_ESM.pdf]

Supplementary Information for

Dolomite luminescence thermochronometry reconstructs the low-temperature exhumation history of carbonate rocks in the central Apennines, Italy

Junjie Zhang[1*], Giorgio Arriga[2, 3], Federico Rossetti[2*], Valentina Argante[1], Dennis Kraemer[4], Mariana Sontag-González[5], Domenico Cosentino[2], Paola Cipollari[2], Sumiko Tsukamoto[1,6]

[1] Leibniz Institute for Applied Geophysics (LIAG), 30655 Hannover, Germany

[2] Dipartimento di Scienze, Università Roma Tre, 00146 Rome, Italy

[3] Archaeology, Environmental Changes, and Geo-Chemistry, Vrije Universiteit Brussel (VUB), Brussels, Belgium

[4] Federal Institute for Geosciences and Natural Resources (BGR), 30655 Hannover, Germany

[5] Institute of Geography, Justus Liebig University of Giessen, 35390 Giessen, Germany

[6] Department of Geosciences, University of Tübingen, 72076 Tübingen, Germany

*corresponding authors: Junjie.Zhang@leibniz-liag.de; federico.rossetti@uniroma3.it

This supplementary file contains:

6 Supplementary Notes

13 Supplementary Figures

5 Supplementary Tables

Supplementary Note 1:

Fig. S1 presents the XRD results of the samples in this study. It proves that the samples are dominated by dolomite.

Fig. S1. X-ray diffraction (XRD) results. The sample details are in Table S1. The samples are dominated by dolomite with trace amount of calcite. Both dolomite and calcite standards are from Mineral Museum of the University of Arizona, with ID numbers of R040030 and R040070 respectively, in the RRUFF database (https://rruff.info/).

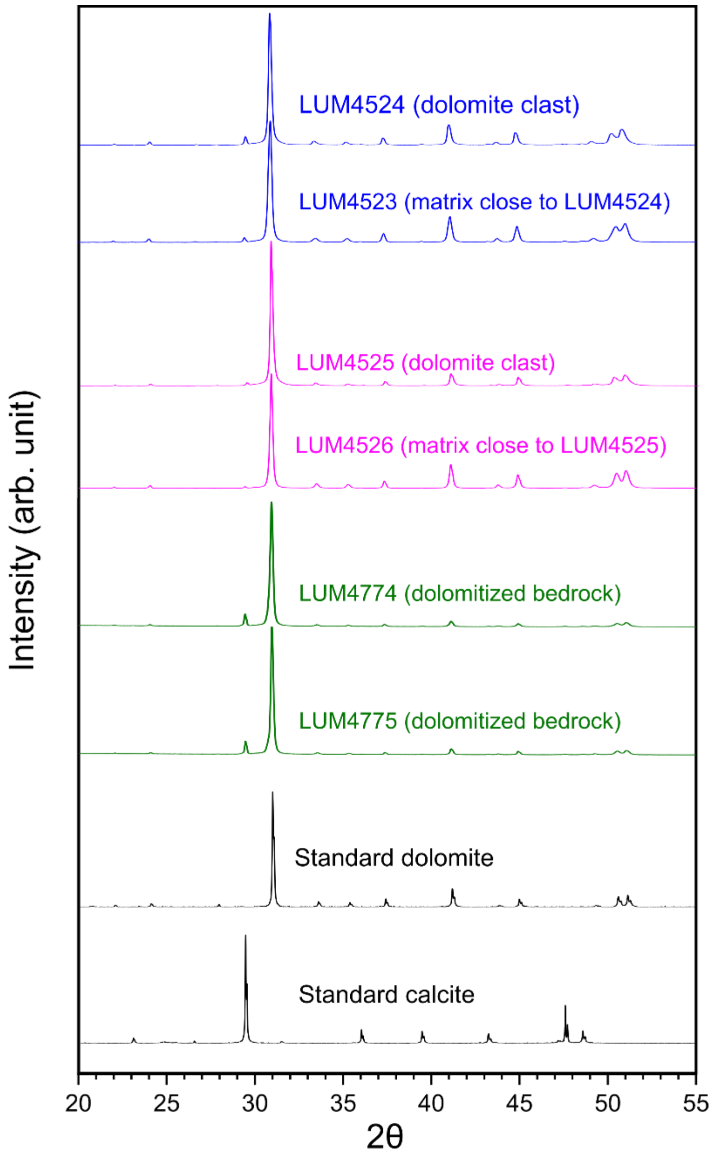

Supplementary Note 2:

Fig. S2 to S4 present the results related to thermoluminescence (TL) measurements.

Fig. S2 shows the TL emission spectrum. Fig. S3 shows the TL signals with peak deconvolution. Fig. S4 gives examples for equivalent dose (D_e) measurements.

Fig. S2. TL emission spectra of a dolomite clast sample (LUM4524) (a, b) and a dolomitized bedrock sample (LUM4774) (c, d). The TL signal of LUM4524 corresponds to a regenerative dose of ~3000 Gy. The TL signal of LUM4774 corresponds to a regenerative dose of ~1000 Gy. Both samples show maximum emission at ~580 nm for three TL peaks. Note that the heating rate used here is 1 °C s^{-1}, and the peak positions shift to lower temperatures compared to the peak positions with a heating rate of 5 °C s^{-1}.

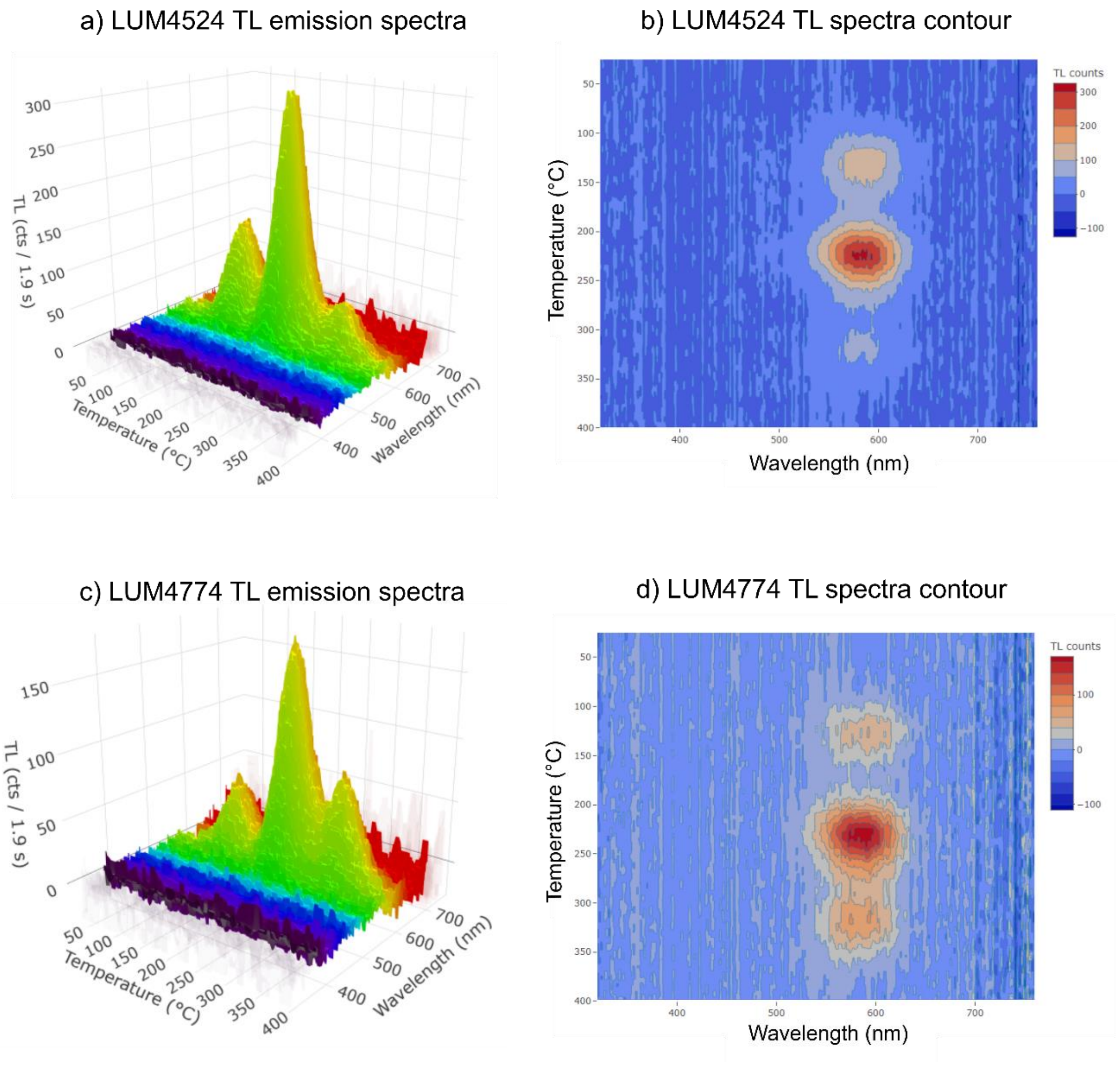

Fig. S3. Peak deconvolution for TL signals. a) Fitting with three peaks for sample LUM4524; b) Fitting with three peaks for sample LUM4773; c) Fitting with four peaks for LUM4524; d) Fitting with four peaks for LUM4773. Note that the fitting with three peaks is not successful, while the fitting with four peaks is successful. The TL signals correspond to a regenerative dose of ~600 Gy. The peak fitting is performed by the Origin2020 software.

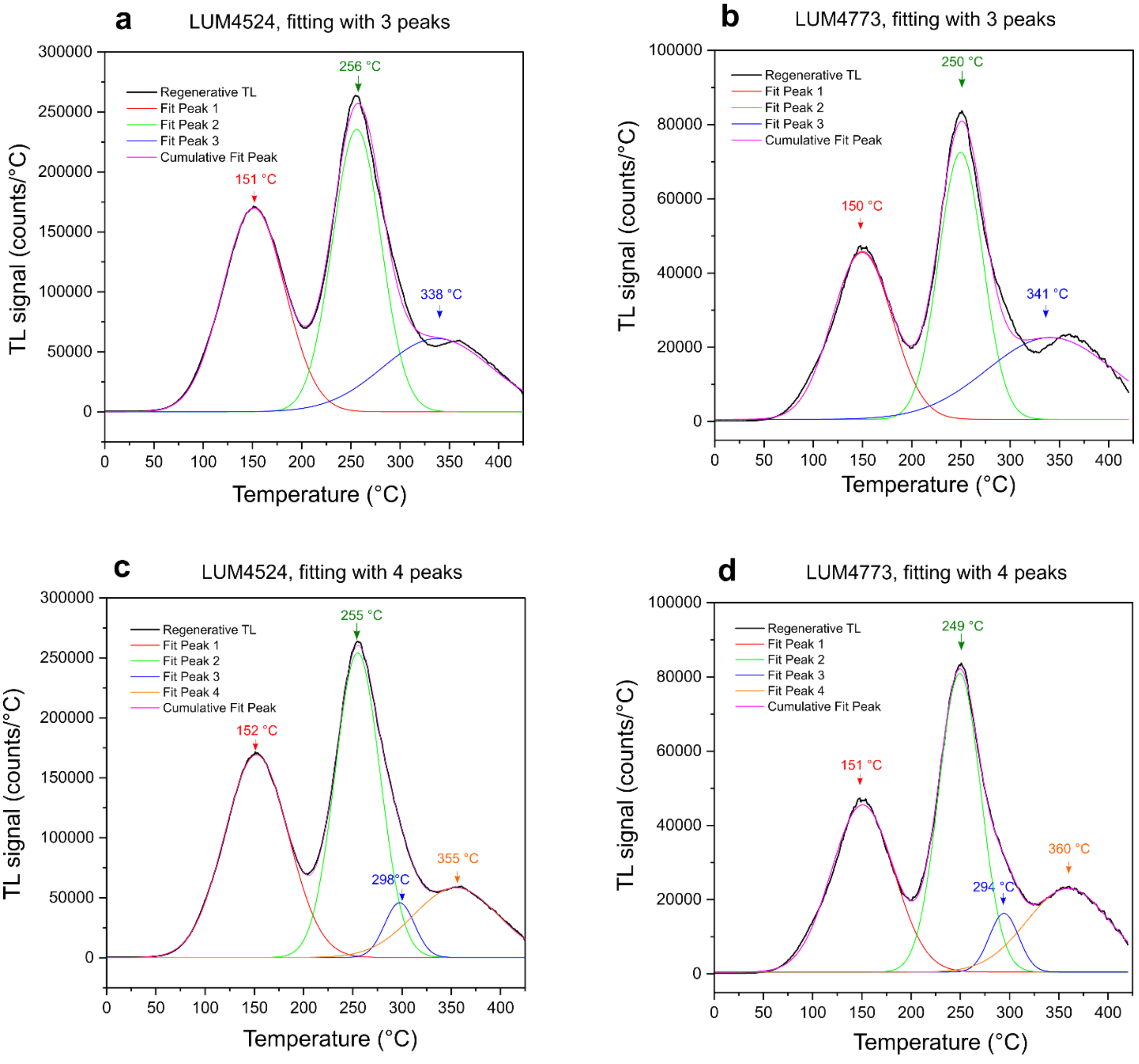

Fig. S4. Examples of MAAD D_e estimation for two clast samples LUM4524 and LUM4773. a, b) TL curves for the natural and natural-additive dose; c, d) dose response curve fitting and D_e estimation. TL curves have been normalized by the 100–180 °C TL signal (response to a dose of 5 Gy) to remove the inter-aliquot variation. Note that the exact peak temperatures change a bit between different samples. For LUM4524, the D_e and D_0 values in figure (c) are slightly different from those in Table 1, as the values in Table 1 have been updated by adding five more aliquots for natural TL signal measurements.

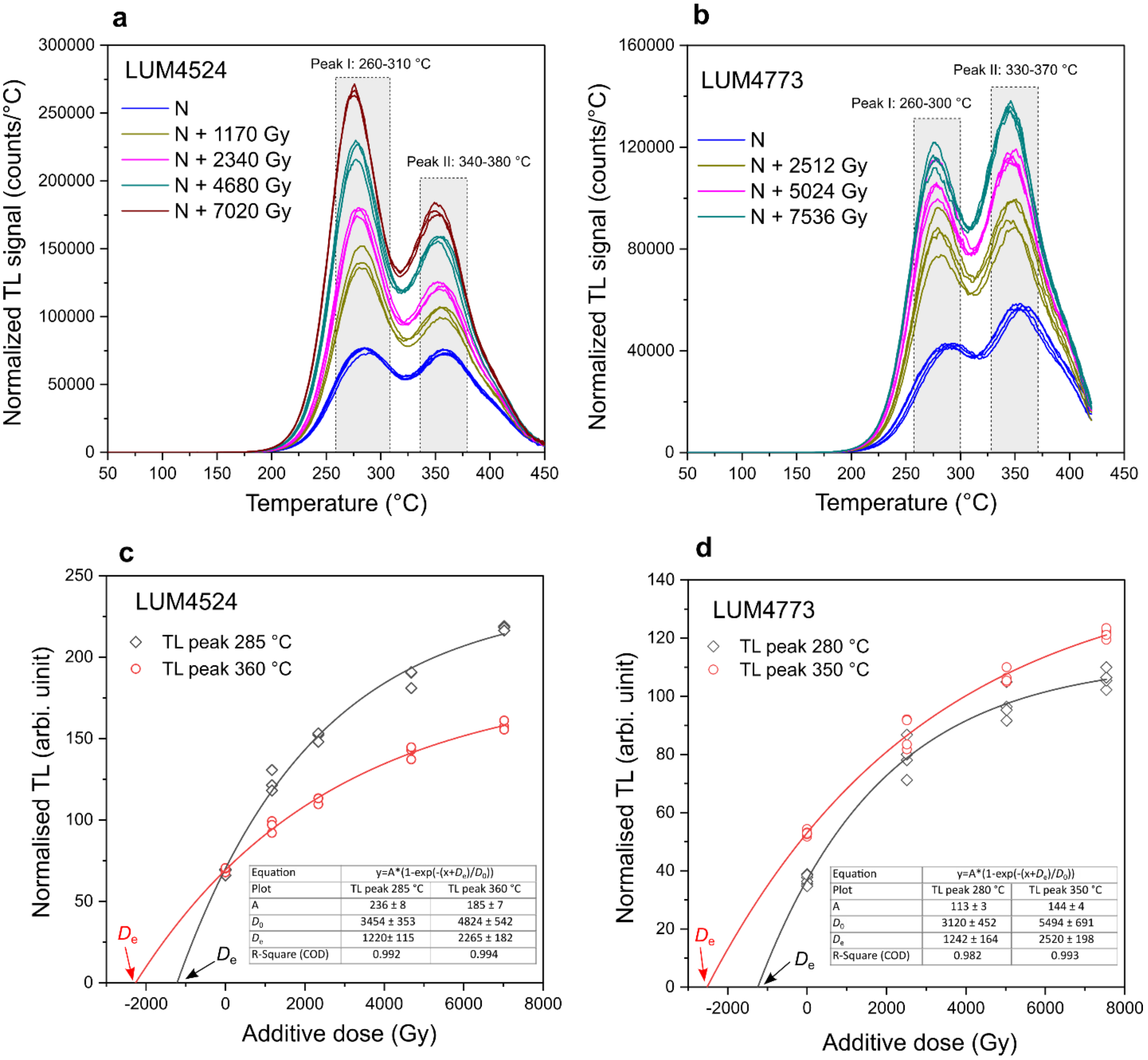

Supplementary Note 3:

Fig. S5 is related to the discussion about the cause of thermoluminescence signal resetting of the dolomite clasts. The rare earth elements and yttrium (REY) data show that the thermoluminescence signal resetting is unlikely due to the alteration/metasomatism by fluids.

Fig. S5. Shale-normalized REY patterns of dolomitized bedrock, fault clasts and cataclastic matrix. Despite the high degree of variation in the overall REY concentrations, all samples show similar REY fractionation features (positive La, Gd and Y anomalies and a typical negative Ce anomaly) which are typical for pristine marine carbonate rocks. The consistent REY signature therefore indicates a closed chemical system without significant metasomatic alteration in the carbonate bedrock during faulting. The REY dataset is normalized to European Shale (EUS).

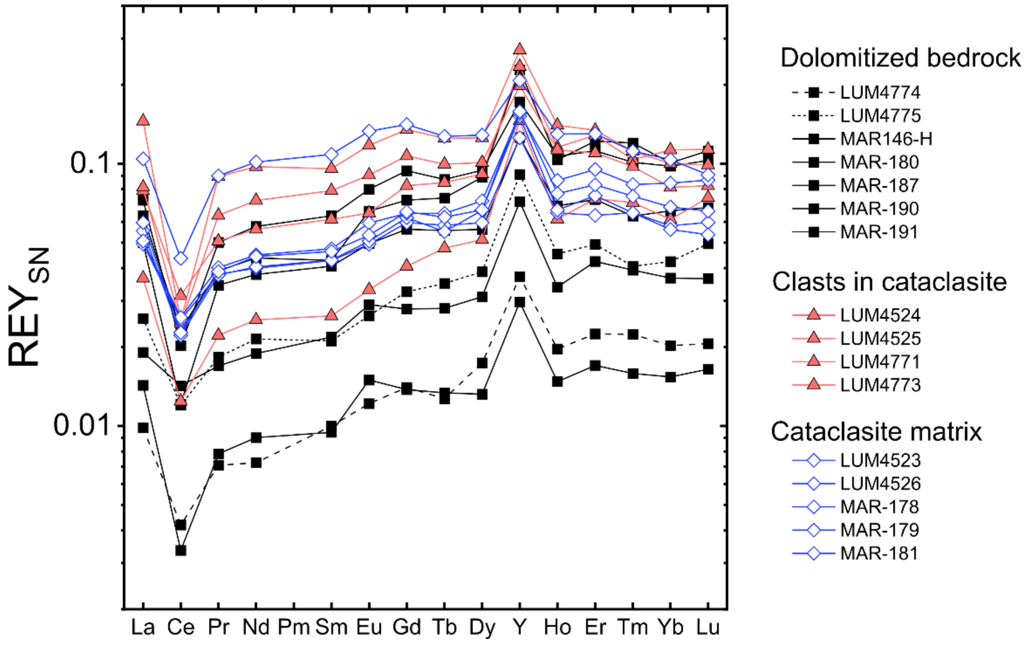

Supplementary Note 4:

Fig S6 to S8 are related to the discussion about the potentials of dolomite TL thermochronometry. Fig. S6 shows the minimum and maximum cooling rates that the dolomite thermochronometry can constrain. Fig. S7 shows how the low thermal stability of the 280 °C TL peak of dolomite will hinder its application in thermochronometry. Fig. S8 shows how the Gaussian distribution of traps (*E* values) will influence the cooling rate modeling.

Fig. S6. Applicable cooling rate range for the dolomite TL thermochronometry. a) Modeling the minimum and maximum applicable cooling rates with signal saturation levels (n/N values) of 85 % and 5 %, respectively, based on the parameters ($\dot{D}$, D_0, E and s) of a clast sample LUM4524. Note that the cooling rate of 20.4 °C Ma^{-1} deduced from the measured n/N is not meaningful, as the TL signals of clasts on the fault damage zone have been reset during a seismic event ~2.5 Ma ago. b) Sample-specific applicable minimum and maximum cooling rates for the six dolomite samples in this study, based on their individual parameters. Generally, the applicable cooling rate range is 2–200 °C Ma^{-1}.

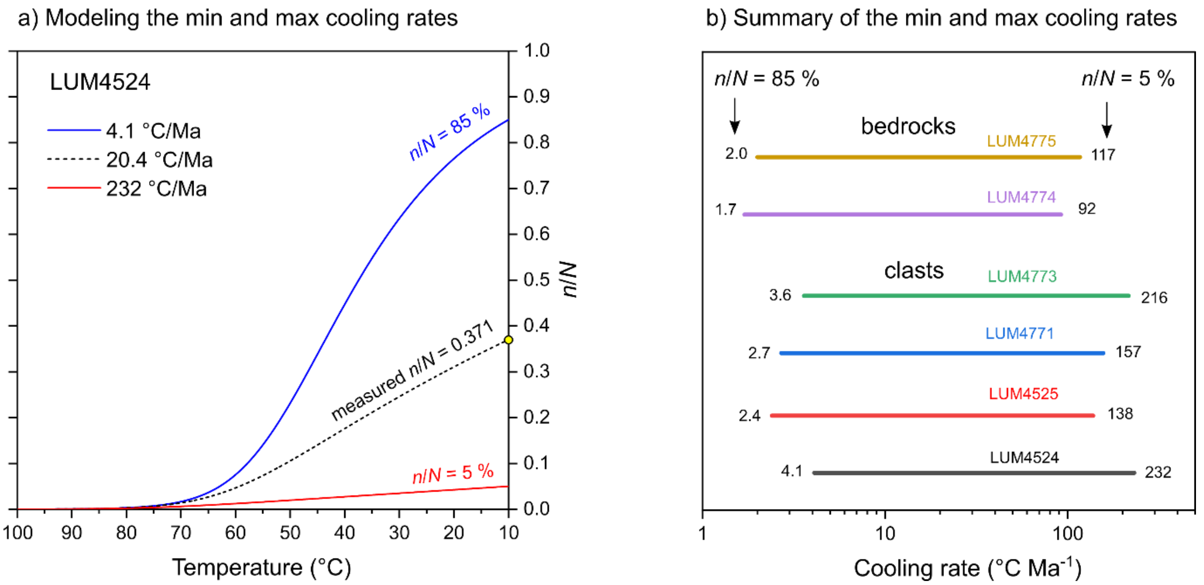

Fig. S7. Cooling history modeling for the 280 °C TL peak (a, b) and 350 °C TL peak (c, d) for one clast sample (LUM4524) and one bedrock sample (LUM4774). The n/N at the thermal equilibrium state, $(n/N)_{ss}$, for each temperature is calculated from $(n/N)_{ss} = \frac{\dot{D}/D_0}{\dot{D}/D_0 + 1/\tau}$. With a cooling rate of 0.01 °C/Ma, the growth of n/N with temperature overlaps with the $(n/N)_{ss}$, because the sample stays at each temperature for 100 Ma which is sufficiently long to reach the thermal equilibrium state. Note that the $(n/N)_{ss}$ for the 280 °C TL peak at the surface temperature (10 °C) can be quite small due to the low thermal stability of the signal.

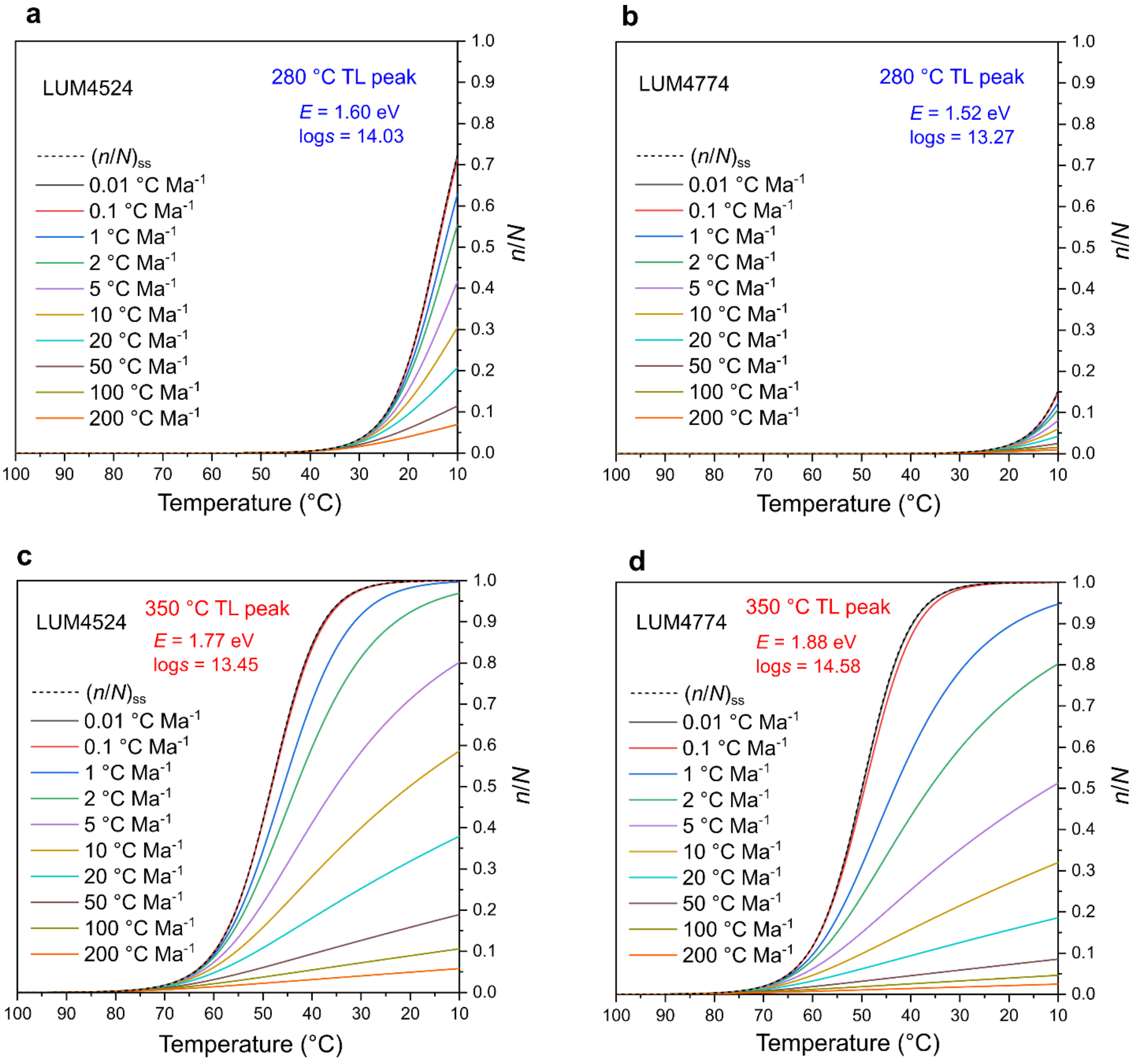

Fig. S8. Cooling rate modeling with and without the assumption of Gaussian distribution of *E* values for the bedrock sample LUM4774. a) Examples for Gaussian distribution of *E* values with different *σE* values (0.05, 0.10 and 0.20 eV) for the 350 °C TL peak (mean *E* = 1.875 eV). b) Inferred cooling rate from the natural *n*/*N* ratio (0.291), with different *σE* values from 0 to 0.3 eV. The cooling rates decrease only slightly with higher *σE* values. Error bars are 1σ. c) Luminescence signal growth under different cooling rates with the single trap model, the same figure as Fig. S7d. d) Luminescence signal growth under different cooling rates with a Gaussian distribution of traps (*σE* = 0.20 eV).

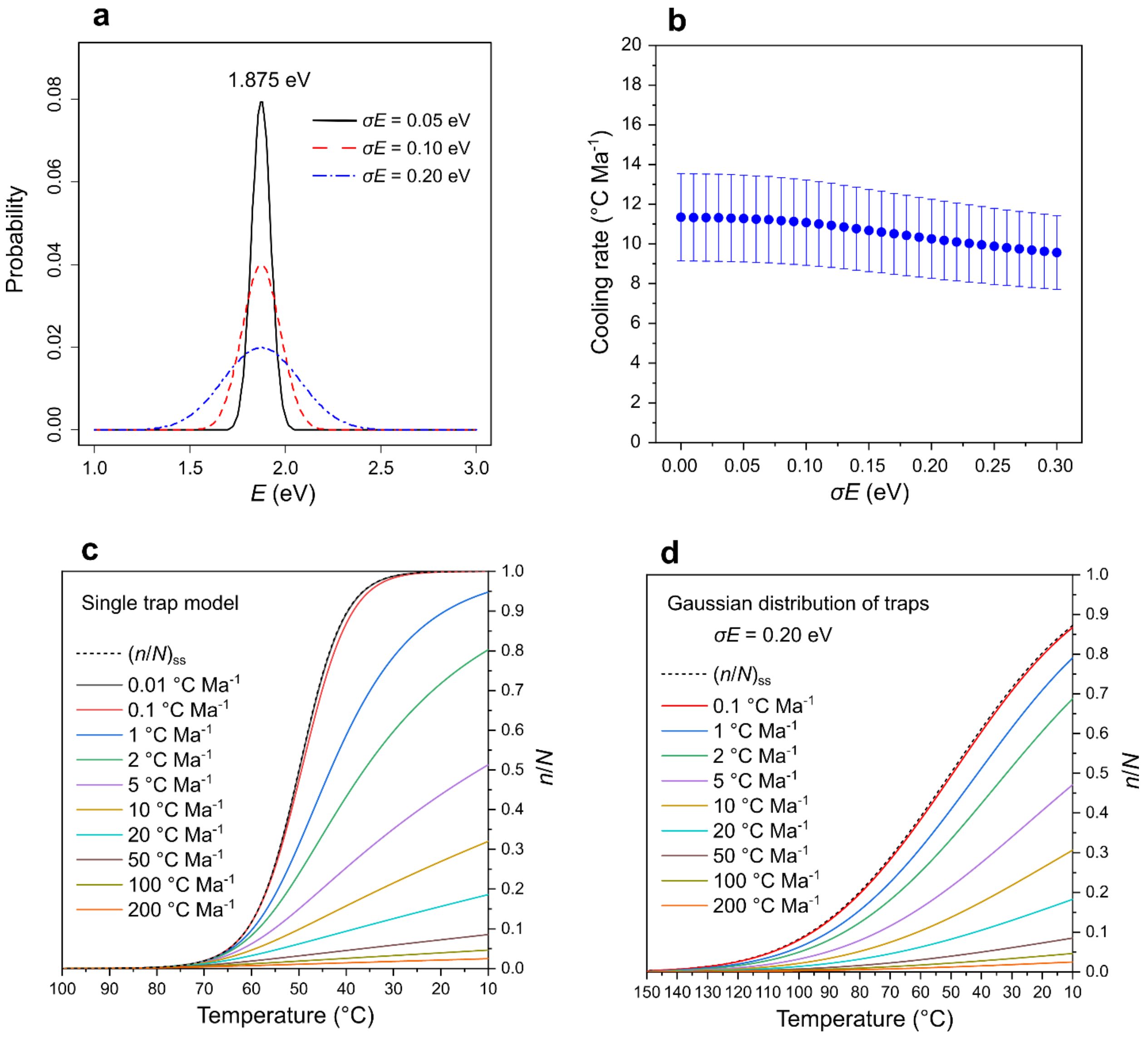

Supplementary Note 5:

Fig S9 to S11 are related to the characteristics of the TL signal of dolomite.

Fig. S9 is about the bleachability of the TL signal, showing the TL signal depletion under solar simulator bleaching. Fig. S10 is about the thermal stability, showing how the activation energy (*E*) and frequency factor (*s*) are estimated by the peak shifting method. Fig. S11 is about the fading rate measurements, showing that the dolomite TL signal has no fading.

Fig. S9. Natural TL curves with no light exposure (aliquot 1 and 2) and natural TL curves after being bleached with a solar simulator (model: Dr. Hönle UVACUBE 400) for 2 hours (aliquot 3 and 4). All TL curves are mass normalized. The 4 aliquots (100–200 µm grains size) are from sample LUM4524.

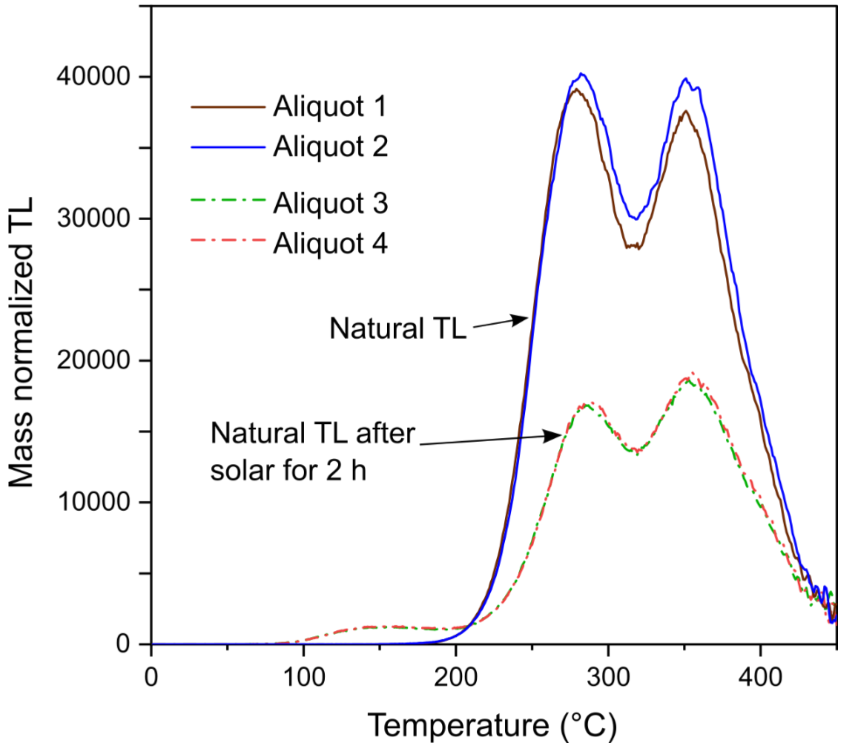

Fig. S10. Examples of thermal lifetime parameters estimation for one aliquot from LUM4524 and one aliquot from LUM4774, respectively. a, c) peak shifting with different heating rates; b, d) linear fitting to estimate the activation energy (E) and frequency factor (s), following the equation: $\ln(T_m^2/\beta)=E/kT_m-\ln(sk/E)$, where β is the heating rate, k is the Boltzmann constant, and T_m is the peak temperature (unit K).

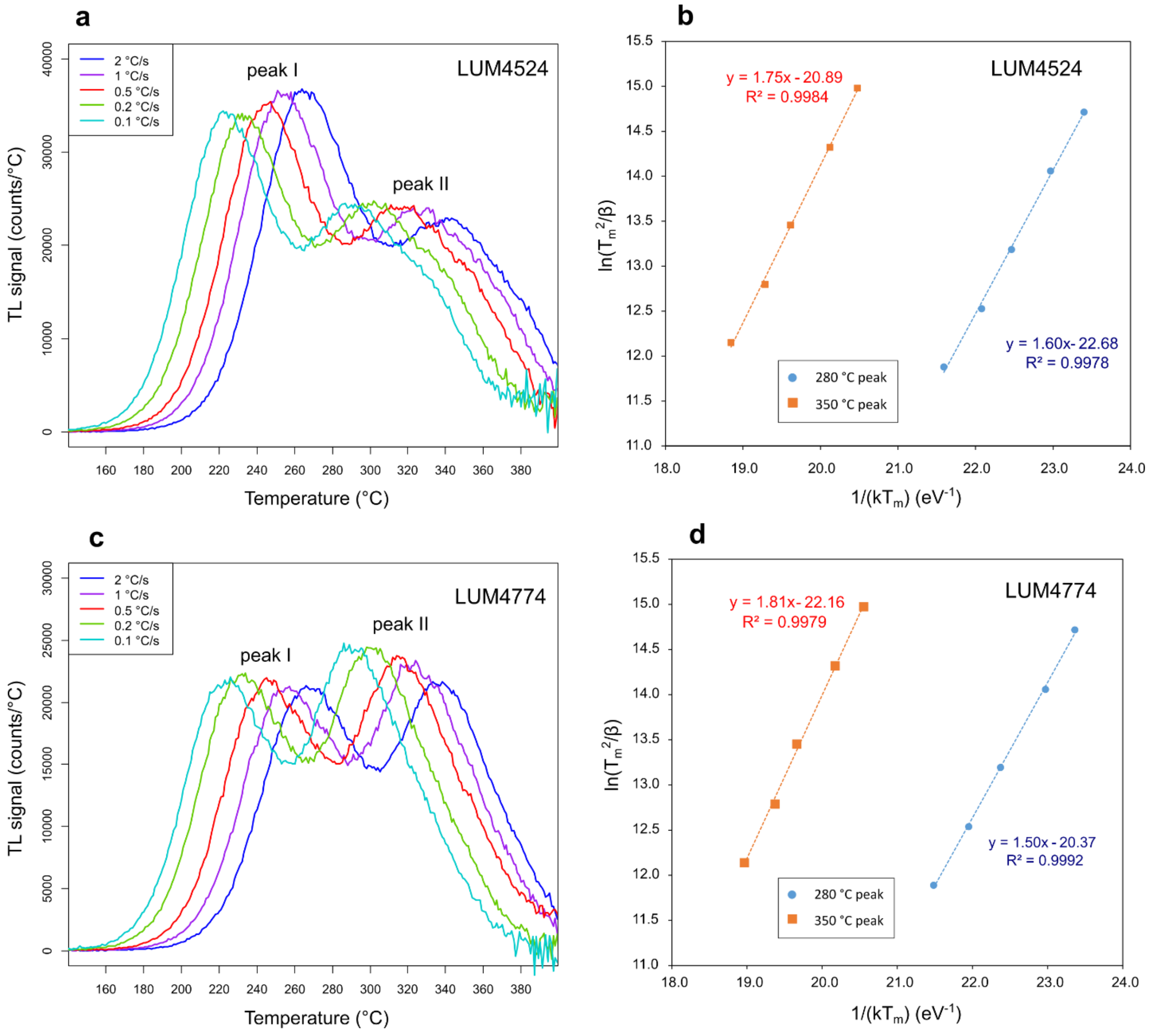

Fig S11. Fading test results. a) TL curves of LUM4524 measured after storage in darkness for 2 weeks (5 aliquots) show no depletion compared to TL curves measured immediately (8 aliquots) after dosing. The given dose is 1200 Gy. b) Fading rates measured for the 350 °C TL peak with the SAR protocol for LUM4524. c) Same as b for LUM4774. All the results show no fading.

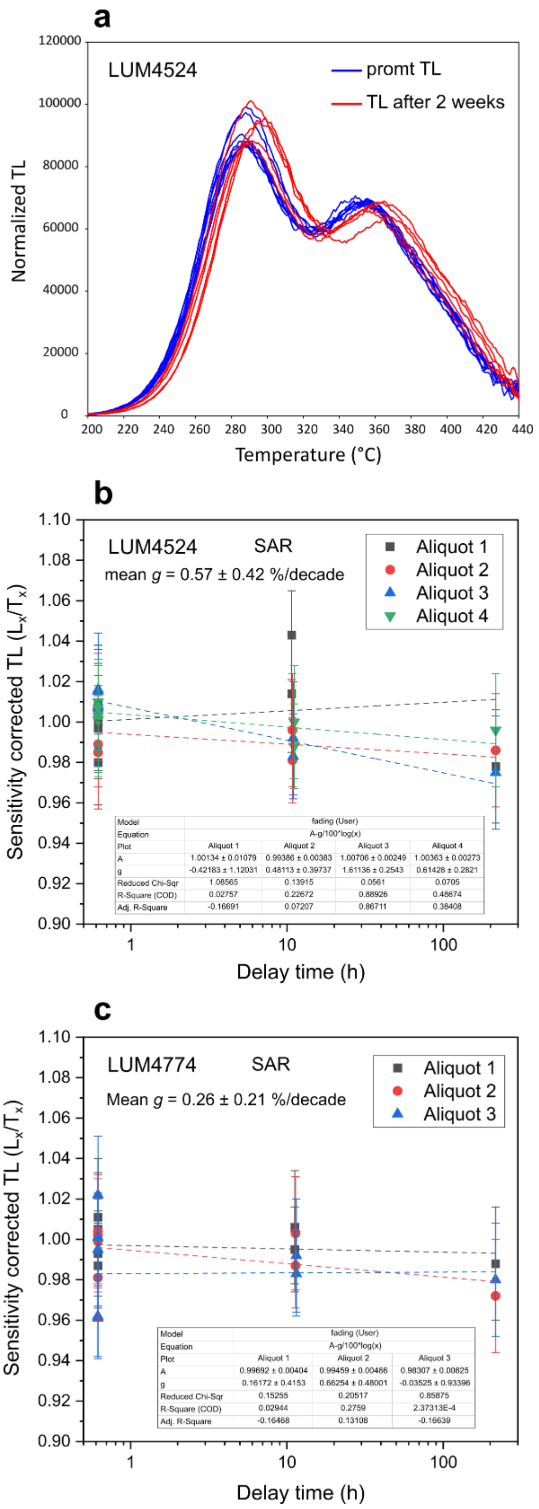

Supplementary Note 6:

Fig. S12 to S13 are related to environmental dose rate calculation.

Fig. S12 is about the alpha efficiency (S_a value) measurements. Fig. S13 shows the cosmic ray dose rates under different depths.

Fig. S12. An example of S_a value measurement with the SAR TL protocol (Table S4) for an aliquot of LUM4524. a) TL curves corresponding to 12 h alpha irradiation and beta doses of 1000 s, 2000 s and 3000 s. b) TL curves of the test dose (beta dose of 1000 s) with different cycles. c) S_a values of TL signals at different temperatures. Note that the TL curves were measured with a heating rate of 1 °C s^{-1}. The TL peaks at ~245 °C and 320 °C correspond to the ~280 °C and 350 °C TL peaks when a heating rate of 5 °C s^{-1} is used. The dose rate of the beta source is 0.133 Gy s^{-1}. The alpha flux rate of the alpha source in University Bordeaux Montaigne is 1.9*10^5 α-particles s^{-1} cm^{-2}.

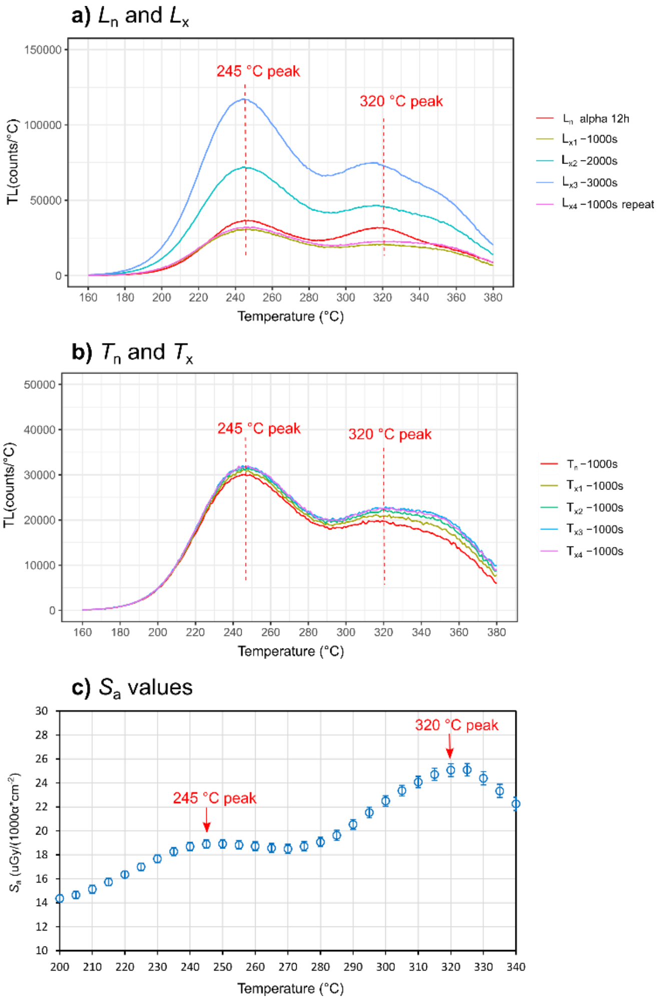

Fig. S13. Cosmic ray dose rates with different depths below the surface at the L'Aquila sampling site. They are calculated based on an elevation of 900 m, a latitude of 42.25° N, and a longitude of 13.28° E. The cosmic ray dose rate is smaller than 0.01 Gy ka^{-1} when the rock is more than 35 m beneath the surface. Considering that the samples in this study were far below the surface for most time during the exhumation history, the contribution of the cosmic ray to the total dose rate is negligible.

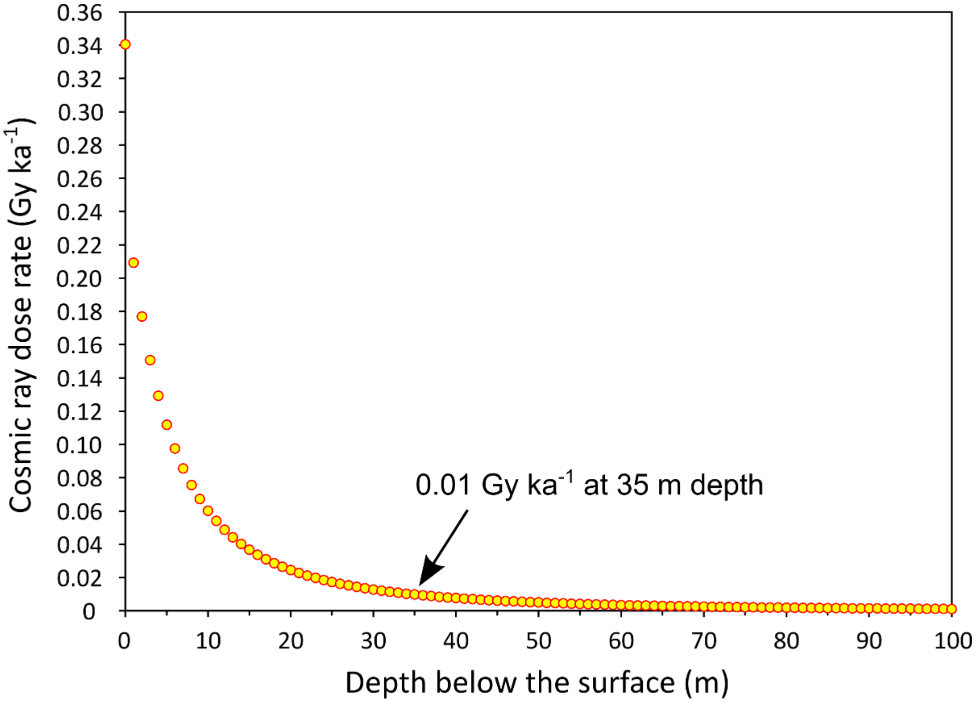

Table S1. Details of sample information. Samples with their ID in bold have been used for TL measurements.

LUM ID	Sample ID	Sample type	Analyses	Latitude (N)	Longitude (E)	Elevation (m)
**LUM4524**	**MAR-2-TL**	Dolomite clast in cataclasite	**TL**, XRD, ICP-MS	42°27'26.1"	13°16'46.7"	889
LUM4523	MAR-1-TL	Matrix in cataclasite	XRD, ICP-MS			
	MAR-181	Matrix in catalcasite	ICP-MS			
**LUM4525**	**MAR-4-TL**	Dolomite clast in cataclasite	**TL**, XRD, ICP-MS	42°27'1.2"	13°17'11.6"	853
**LUM4771**	**MAR-7-TL**	Dolomite clast in cataclasite	**TL**, ICP-MS			
LUM4526	MAR-5-TL	Matrix in cataclasite	XRD, ICP-MS			
	MAR-179	Matrix in cataclasite	ICP-MS			
**LUM4773**	**MAR-18**	Dolomite clast in cataclasite	**TL**, ICP-MS	42°27'7.79"	13°17'2.94"	868
	MAR-178	Matrix in cataclasite	ICP-MS			
**LUM4774**	**MAR-29**	Dolomitized bedrock	**TL**, XRD, ICP-MS	42°26'50.02"	13°17'38.51"	928
**LUM4775**	**MAR-30**	Dolomitized bedrock	**TL**, XRD, ICP-MS	42°26'53.07"	13°17'25.66"	875
	MAR146-H	Dolomitized Bedrock	ICP-MS	42°26'36.83"	13°18'11.22"	1007
	MAR-180	Dolomitized Bedrock	ICP-MS	42°27'1.71"	13°17'13,31"	874
	MAR-187	Dolomitized Bedrock	ICP-MS	42°27'8.04"	13°18'22.91"	1413
	MAR-190	Dolomitized Bedrock	ICP-MS	42°27'2.12"	13°19'10.67"	1246
	MAR-191	Dolomitized Bedrock	ICP-MS	42°26'55.36"	13°19'8.15"	1239

Table S2. The modified multiple-aliquot additive-dose (MAAD) protocol used for D_e measurement. The heating rates are 5 °C s^{-1}. The T_x is the low-temperature TL signal (100–180 °C) of a small dose of 5 Gy. It will be used to normalize the 350 °C TL peak signal (L_x) to reduce the inter-aliquot variation. This normalization method is equivalent to mass normalization.

Step	Treatments	Signal
1	Give a small dose (5 Gy)	
2	Heat to 200 °C	T_x
3	Give additive dose (0–8000 Gy)	
4	Preheat to 260 °C	
5	TL to 450 °C with background subtraction	L_x

Table S3. Thermal kinetic parameters (E, s) and lifetimes at 10 °C (τ_{10}) and 20 °C (τ_{20}) for the TL peaks at ~350 °C and 280 °C (with a heating rate of 5 °C s^{-1}). For each sample, three aliquots were applied for peak shifting measurements. The mean E was calculated from $(E_1+E_2+E_3)/3$, and the mean s from $(s_1*s_2*s_3)^{1/3}$. Note that the parameters for the 280 °C TL peak may not be meaningful, as this peak is a combination of two peaks. For the 280 °C TL peak, the signal saturation levels at the thermal equilibrium state, the $(n/N)_{ss}$ values, are calculated at temperatures of 10 °C and 20 °C. Note that, for the 350 °C TL peak, these $(n/N)_{ss}$ values are 1 at temperatures of 10 °C and 20 °C, due to the long lifetimes.

Sample ID	Sample type	350 °C TL peak				280 °C TL peak					
		E (eV)	s (s^{-1})	τ_{10} (Ma)	τ_{20} (Ma)	E (eV)	s (s^{-1})	τ_{10} (Ma)	τ_{20} (Ma)	$(n/N)_{ss\_10}$	$(n/N)_{ss\_20}$
LUM4524	Clast	1.77	2.81E+13	29735	2520	1.60	1.08E+14	7.56	0.812	0.72	0.22
LUM4525	Clast	1.78	4.30E+13	36827	3054	1.53	4.34E+13	1.03	0.122	0.18	0.03
LUM4771	Clast	1.77	4.20E+13	23973	2019	1.52	2.31E+13	1.72	0.205	0.28	0.04
LUM4773	Clast	1.71	9.88E+12	8306	762	1.58	8.22E+13	5.35	0.586	0.68	0.19
LUM4774	Bedrock	1.88	3.76E+14	188749	13748	1.52	1.85E+13	1.68	0.202	0.15	0.02
LUM4775	Bedrock	1.80	8.67E+13	44587	3587	1.53	2.04E+13	2.53	0.298	0.17	0.02

Table S4. SAR TL protocol for S_a value measurements.

Step	Treatments	Signal
1	Alpha irradiation 12 h for the first cycle, beta irradiation for other cycles	
2	Preheat to 260 °C (5 °C s^{-1})	
3	TL to 400 °C (1 °C s^{-1})	L_x
4	Test dose (beta irradiation 1000 s)	
5	Preheat to 260 °C (5 °C s^{-1})	
6	TL to 400 °C (1 °C s^{-1})	T_x
7	Return to step 1	

Table S5. U, Th, K concentrations, alpha efficiency factors (S_a value) and dose rates. Errors are 1σ. For clast samples LUM4771 and LUM4773, the mean S_a value of clast samples LUM4524 and LUM4525 is used for dose rate calculation.

Sample ID	Sample type	U (ppm)	Th (ppm)	K (ppm)	S_a (μGy/(1000α*cm^{-2}))	Dose rate (Gy ka^{-1})			
						Alpha	Beta	Gamma	Total
LUM4524	Clast	1.076 ± 0.005	0.249 ± 0.006	268 ± 14	27.6 ± 2.7	0.525	0.185	0.139	0.849 ± 0.056
LUM4525	Clast	0.874 ± 0.011	0.197 ± 0.005	161 ± 6	26.7 ± 2.0	0.412	0.146	0.111	0.669 ± 0.037
LUM4771	Clast	0.671 ± 0.013	0.066 ± 0.003	24.3 ± 1.2	27.2 ± 1.7	0.311	0.102	0.079	0.491 ± 0.026
LUM4773	Clast	1.403 ± 0.028	0.129 ± 0.007	30.3 ± 1.5	27.2 ± 1.7	0.649	0.211	0.164	1.024 ± 0.055
LUM4774	Bedrock	0.469 ± 0.009	0.022 ± 0.001	20.8 ± 1.0	15.9 ± 1.6	0.125	0.071	0.054	0.250 ± 0.015
LUM4775	Bedrock	0.446 ± 0.009	0.036 ± 0.002	22.4 ± 1.1	15.7 ± 1.7	0.119	0.068	0.052	0.239 ± 0.015
